# A prospective study to assess the value of MMP-9 in improving the appropriateness of urgent referrals for colorectal cancer

**DOI:** 10.1186/1471-2407-6-251

**Published:** 2006-10-23

**Authors:** Angela V Ryan, Sue Wilson, Michael JO Wakelam, Sally A Warmington, Janet A Dunn, Richard FD Hobbs, Ashley Martin, Tariq Ismail

**Affiliations:** 1 Department of Primary Care and General Practice, University of Birmingham, Birmingham B15 2TT, UK; 2 Cancer Research UK Institute for Cancer Studies, University of Birmingham, Birmingham B15 2TT, UK; 3 Health Sciences Research Institute, University of Warwick, Gibbett Hill Campus, Coventry CV4 7AL, UK; 4 University Hospital Birmingham Foundation NHS Trust, Queen Elizabeth Hospital, Birmingham B15 2TH, UK

## Abstract

**Background:**

Bowel cancer is common and is a major cause of death. Most people with bowel symptoms who meet the criteria for urgent referral to secondary care will not be found to have bowel cancer, and some people who are found to have cancer will have been referred routinely rather than urgently. If general practitioners could better identify people who were likely to have bowel cancer or conditions that may lead to bowel cancer, the pressure on hospital clinics may be reduced, enabling these patients to be seen more quickly. Increased levels of an enzyme called matrix metalloproteinase 9 (MMP-9) have been found to be associated with such conditions, and this can be measured from a blood sample. This study aims to find out whether measuring MMP-9 levels could improve the appropriateness of urgent referrals for patients with bowel symptoms.

**Methods:**

People aged 18 years or older referred to a colorectal clinic will be asked to complete a questionnaire about symptoms, recent injuries or chronic illnesses (these can increase the level of matrix metalloproteinases) and family history of bowel cancer. A blood sample will be taken from people who consent to take part to assess MMP-9 levels, and the results of examination at the clinic and/or investigations arising from the clinic visit will be collected from hospital records. The accuracy of MMP-9 will be assessed by comparing the MMP-9 level with the resulting diagnosis. The combination of factors (e.g. symptoms and MMP-9 level) that best predict a diagnosis of malignancy (invasive disease or polyps) will be determined.

**Discussion:**

Although guidelines are in place to facilitate referrals to colorectal clinics, symptoms alone do not adequately distinguish people with malignancy from people with benign conditions. This study will establish whether MMP-9 could assist this process. If this were the case, measurement of MMP-9 levels could be used by general practitioners to assist in the identification of people who were most likely to have bowel cancer or conditions that may lead to bowel cancer, and who should, therefore, be referred most urgently to secondary care.

## Background

Bowel cancer is common and is a major cause of death. It is the third and second most common cancer in men and women, respectively, in England, and there are over 28000 new cases in England, and over 14000 deaths in England and Wales each year.[1,2] Survival is associated with stage of disease at diagnosis.[3] Benefits, in terms of improved survival, improved quality of life and reduced treatment costs, could be accrued by earlier diagnosis. It is, therefore, important to concentrate on improving early detection of disease.

Guidelines have been introduced to facilitate appropriate primary care referrals for people with suspected cancer.[4,5] Most people with bowel symptoms who meet the criteria for urgent referral will not, however, be found to have bowel cancer.[6] Also, some people who are subsequently found to have cancer will have been referred routinely rather than urgently. If people who were most likely to have bowel cancer or conditions that may lead to bowel cancer could be identified and referred most urgently, the pressure on hospital clinics could be reduced, enabling these patients to be seen more quickly.

Available diagnostic tests for bowel cancer include colonoscopy and flexible sigmoidoscopy. These tests are invasive with an element of risk, and they usually need to be done in hospital. A simple and acceptable test that could be used by general practitioners could save many people from having unnecessary and invasive procedures. Increased levels of an enzyme called matrix metalloproteinase 9 (MMP-9) have been found to be associated with bowel cancer and conditions that may lead to bowel cancer,[7-9] and this can be measured from a blood sample.

### Study aim

To find out whether a new blood test (MMP-9) could improve the appropriateness of urgent referrals for patients with bowel symptoms.

### Study objectives

To describe quality of life, symptom profiles and anxiety in new referrals to colorectal clinics. To take a blood sample from each person to measure their MMP-9 level. To review each person's hospital records to find out if they were diagnosed with bowel cancer or polyps. To determine the combination of factors (e.g. symptoms and MMP-9 level) that best predict whether a person has bowel cancer or is at high risk of developing the disease.

## Methods

### Study design

Evaluation of the accuracy of a diagnostic test: accuracy of MMP9 will be assessed by comparison with the results of examinations and investigations undertaken at, or prompted by, the colorectal clinic visit.

### Population to be studied

People will be eligible if they are aged 18 years or older and are a new referral (routine or urgent) to the colorectal clinic at University Hospital Birmingham NHS Foundation Trust during the study period.

### Selection of participants

Potential participants will be identified using the Trust appointment system. An information leaflet, cover letter and questionnaire will be sent to each eligible person approximately two weeks before their clinic appointment. The questionnaire asks about symptoms, recent injuries or chronic illnesses (as these can also increase the level of matrix metalloproteinases) and whether the respondent or any close family members have had bowel cancer. People who may want to take part will be asked to come to the clinic at their appointment time with the completed questionnaire. The study receptionist will ask potential participants as they arrive at the clinic whether they would like to speak with a researcher about the study. Those considering participation will be asked to complete two short survey instruments as they wait: the SF-12 assesses overall health status[10] and the State-Trait Anxiety Inventory assesses temporary and long-term anxiety [11].

To ensure that people who attend the clinic at short notice also have the opportunity to participate, we will put a poster in the waiting room of the clinic. The poster will outline in layman's terms the aim of the study and what taking part would entail and ask that patients tell the study receptionist if they are interested in participating. Interested patients will be asked to read the information leaflet and, if they still wish to participate, to complete the questionnaires.

### Informed consent

A trained researcher will speak with each potential participant and seek informed consent. Private rooms at the colorectal clinic will be available for this purpose. Working to a script to avoid interviewer bias and ensure that all important points are covered, the researcher will outline why the study is being done, what taking part would mean, and the associated risks. Potential participants will be able to ask questions at any point. People who are unable to understand why the study is being done or what taking part would mean will be excluded. People who have not been excluded and who decide that they would like to take part in the study will be asked to sign the consent form. The survey instruments and questionnaire will be collected from consented participants and checked. Non-directive assistance will be provided for any unanswered questions.

### Exclusion criteria

People who are unwilling to give informed consent or who are unable to understand why the study is being done or what taking part would mean will be excluded.

### Clinical evaluations, laboratory tests and follow-up

The researcher will take a blood sample for MMP-9 estimation from consented participants. Each person will then be taken back into the waiting room for their clinic appointment. At the end of each session, all blood samples will be taken to the Institute of Cancer Studies at the University of Birmingham, where they will be analysed. To reduce bias, the technician doing the analysis will not be aware of the participants' symptoms or diagnoses.

The results of each participant's examination at the colorectal clinic and any investigations arising from the clinic visit will be collected from their hospital records using a standard proforma. This will be done after a gap of at least two months to allow relevant investigations to be completed and documented. Any missing information will be sought from the consultant who cared for the person. Participants without a diagnosis of colorectal cancer will be flagged and followed-up via the NHS Central Register.

### Procedures for handling data

All data will be entered onto password-protected databases. Passwords will be changed regularly and only known by core research staff. Personal data will be stored in a separate database with a different password from other data. Participants will also be allocated a unique study number that will be used when possible, and the results of the study will only be presented so that no single person can be identified. Members of the research team from the University will hold honorary contracts with the Trust, and all members of the research team are trained in procedures related to confidentiality.

### Sample size estimate

**Figure 1:**
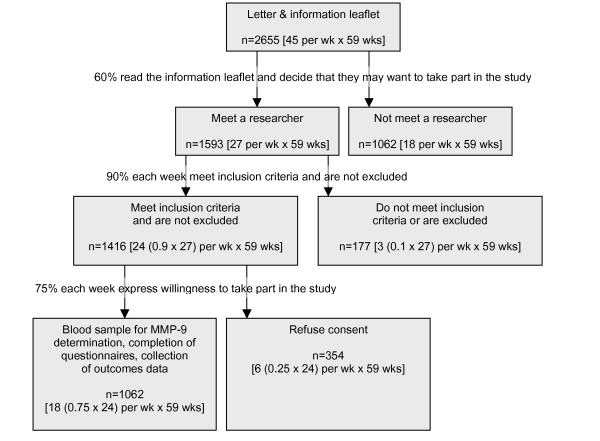
Research plan and study design.

The sensitivity of a diagnostic test is important when a disease has serious consequences, for example colorectal neoplasia. We have, therefore, based our calculation on the confidence interval and precision with which we want to estimate the sensitivity of MMP-9. We obtained a sensitivity of 99% in a pilot study of 46 normal volunteers and 300 patients attending a specialist clinic. To estimate this sensitivity with +/-2.5% precision and a 95% confidence interval, we need blood samples from 60 people with colorectal neoplasia. Based on studies where asymptomatic populations were screened,[12-14] we conservatively estimate the prevalence of colorectal neoplasia as 6%. Using this prevalence, the overall sample size would be 60 × 100/6 = 1000 people, and 940 people would not have colorectal neoplasia. This would allow us to estimate a specificity of 63% (as obtained in the pilot study) with +/-4.0% precision and a 95% confidence interval. This precision can be improved to +/-3.0% by increasing the number of people without colorectal neoplasia to 994. The overall sample size would then be (994 × 100/94 =) 1057 people, and we estimate that it will take about 60 weeks to complete recruitment (figure 1). We would expect (1057 - 994 =) 63 of these people to have colorectal neoplasia, which would still allow us to estimate a sensitivity of 99% with +/-2.5% precision and a 95% confidence interval.

### Analysis

#### Accuracy of MMP-9

ROC curve analyses will be undertaken to permit decisions regarding the best choice of cut-off points (MMP-9) to achieve specified levels of misclassification (either overall misclassification or maximum negative predictive value). Supplementary analyses of appropriate subgroups (initially by logistic linear regression) will determine whether different cut-offs are appropriate in different age groups or, for example, symptom profiles. The sensitivity, specificity, positive predictive value and negative predictive value of the test will be calculated. Confidence intervals will be provided for all summary statistics.

#### Predicting those patients who will have a diagnosis of cancer

The purpose of the analysis is to determine whether the routine estimation of MMP-9 would better enable identification of those patients with significant pathology. Independent variables will include age, sex, socio-economic status, symptoms, whether close family members have had bowel cancer, and MMP-9 level. Given the categorical nature of the outcome (no disease, polyps, invasive disease), discriminant analysis will be used to determine the combination of factors that best predicts a diagnosis of malignancy (invasive disease/polyps).

### Research governance

The dignity, rights, safety and well being of participants will be given priority at all times. All relevant agencies will be made fully aware of the research and all relevant professionals will be asked to take responsibility for clinical care. Adverse event monitoring will be undertaken. The chief and key investigators will form a trial management group, which will meet regularly to review the conduct of the research and fidelity to the trial protocol, including uptake and progress towards the recruitment target. The researchers will record reasons for exclusion and any notable events. They will meet regularly to review these practical issues, and a representative will report back at the trial management group meeting.

#### Ethical approval

This study has been approved by North Birmingham Local Research Ethics Committee: reference 04/Q2704/29.

## Discussion

Bowel cancer is common and is a major cause of death. Although guidelines are in place to facilitate referrals to colorectal clinics, symptoms alone may not adequately distinguish people with malignancy from people with benign conditions. If general practitioners could identify people who were most likely to have bowel cancer or conditions that may lead to bowel cancer, the pressure on hospital clinics could be reduced, enabling these patients to be seen more quickly. A blood test is likely to be acceptable to most people. Increased levels of an enzyme called matrix metalloproteinase 9 (MMP-9) have been found to be associated with bowel cancer and conditions that may lead to bowel cancer, and this can be measured from a blood sample. This study will establish whether measurement of MMP-9 levels from a blood sample could improve the appropriateness of urgent referrals for patients with bowel symptoms.

## Competing interests

The author(s) declare that they have no competing interests.

## Authors' contributions

SW, TI, MO and AR designed the study. MO and AM are responsible for MMP9 estimation, TI and RH have responsibility for clinical, and JD statistical, aspects of the study. SAW has responsibility for data collection and validation. All authors read and approved the final manuscript.

## Pre-publication history

The pre-publication history for this paper can be accessed here:
